# Prognostic and immunological role of FDX1 in pan-cancer: an in-silico analysis

**DOI:** 10.1038/s41598-023-34752-1

**Published:** 2023-05-16

**Authors:** Ziqiang Liu, Jinfeng Miao

**Affiliations:** 1 General Medicine Department, Taikang Tongji (Wuhan) Hospital, Wuhan, 430050 China; 2 Department of Neurology, Tongji Hospital, Tongji Medical College, Huazhong University of Science and Technology, Wuhan, 430030 China

**Keywords:** Bioinformatics, Gene expression analysis, Cancer, Immunology, Biomarkers, Oncology

## Abstract

Previous research has demonstrated that ferredoxin 1 (FDX1) contributes to the accumulation of toxic lipoylated dihydrolipoamide S-acetyltransferase (DLAT) and results in cuproptotic cell death. However, the role that FDX1 plays in human cancer prognosis and immunology is still not well understood. The original data was obtained from TCGA and GEO databases and integrated using R 4.1.0. The TIMER2.0, GEPIA, and BioGPS databases were used to explore FDX1 expression. The impact of FDX1 on prognosis was analyzed using the GEPIA and Kaplan–Meier Plotter databases. External validation will be performed using the PrognoScan database. FDX1 expression in different immune and molecular subtypes of human cancers was evaluated using the TISIDB database. The correlation between FDX1 expression and immune checkpoints (ICP), microsatellite instability (MSI), and tumor mutational burden (TMB) in human cancers was analyzed using R 4.1.0. The TIMER2.0 and GEPIA databases were used to study the relationship between FDX1 expression and tumor-infiltrating immune cells. With the c-BioPortal database, we investigated the genomic alterations of FDX1. Pathway analysis and assessment of the sensitivity potential of FDX1-related drugs were also performed. Using the UALCAN database, we analyzed the differential expression of FDX1 in KIRC (kidney renal clear cell carcinoma) with different clinical features. Coexpression networks of FDX1 were analyzed using LinkedOmics. In general, FDX1 was expressed differently in different types of cancer in humans. Expression of FDX1 was strongly correlated with patient prognosis, ICP, MSI, and TMB. FDX1 was also participated in immune regulation and the tumor microenvironment. Coexpression networks of FDX1 were primarily involved in oxidative phosphorylation regulation. Pathway analysis revealed that the expression of FDX1 was correlated to cancer-related and immune-related pathways. FDX1 has the potential to serve as a biomarker for pan-cancer prognosis and immunology, as well as a novel target for tumor therapy.

## Introduction

Cuproptosis is a novel form of regulated cell death characterized by copper-mediated aggregation of mitochondrial lipoylated proteins and destabilization of Fe-S cluster proteins^1^. Although research on the relationship between cuproptosis and cancer is still emerging, there is mounting evidence suggesting that copper is involved in the etiology, genesis, severity, and progression of various types of cancer, such as breast cancer, head and neck cancer, and endometrial cancer^2–6^.

Copper is known to promote angiogenesis, a critical factor in tumor growth and dissemination^7,8^. It suggested that cuproptosis might be used as a natural and promising therapy against cancer. However, the expression patterns and prognostic values of key cuproptosis regulators, particularly their association with immune infiltration, remain to be elucidated.

Ferredoxin 1 (FDX1), a critical metabolism-related gene, has been implicated in the regulation of cuproptosis via its involvement in the destabilization of Fe-S cluster proteins^9^. Additionally, FDX1 has been linked to cancer progression, prognosis, and immune response across a range of cancer types. FDX1's dual role in cuproptosis and cancer biology increases the likelihood of identifying novel therapeutic targets and prognostic biomarkers through its study.

In lung adenocarcinoma (LUAD), FDX1 knockdown did not inhibit tumor cell growth or induce apoptosis or abnormal cell cycle distribution^10^. Nevertheless, FDX1 has been found to enhance ATP production and is closely associated with glucose metabolism, fatty acid oxidation, and amino acid metabolism. These observations suggest that FDX1 may have unique functions in cancer biology warranting further investigation.

Although previous bioinformatics analyses have proposed the potential value of FDX1 in cancer diagnosis and treatment, most studies lack external validation or are only validated across tumor cell lines^11–14^. In the present study, we conducted a comprehensive analysis to examine the role of FDX1 in human cancer prognosis and immunology. We also investigated the relationship between FDX1 expression and immune subtypes, molecular subtypes of various cancer types, immunobiomarkers in the tumor microenvironment (TME), and effective small molecule drugs. Finally, we investigated the effect of FDX1 expression in KIRC (kidney renal clear cell carcinoma) to verify our findings in human cancer. The objective of this study was to evaluate the potential of FDX1 in anticancer immunotherapy in human cancer, offering insights into a novel approach to cancer treatment.

## Methods

### Data and software availability

All original data were downloaded from the Gene Expression Omnibus (GEO) (https://www.ncbi.nlm.nih.gov/geo/) and Cancer Genome Atlas (TCGA) (https://cancergenome.nih.gov/) databases. GEO is an international repository for gene expression data and functional genomics data. TCGA consists of more than 20,000 primary cancer samples over 33 cancer types^15^. We used R 4.1.0 for the integration of the original data and verification of the website database results.

### The expression of FDX1 in human cancers is analysed in three databases

On the basis of the TIMER2.0 (https://cistrome.shinyapps.io/timer/) and GEPIA (http://gepia2.cancer-pku.cn/#analysis) databases, FDX1 expression was compared between human cancers and paired normal tissues^16,17^. The TIMER2.0 database contains 10,897 samples across 32 cancer types from TCGA. GEPIA is an online database, with data on tumors and normal tissues obtained from the TCGA database. Additional tumors were analyzed that were not covered in TIMER2.0 databases. FDX1 expression profiles in various cancer cell lines and paired normal cell lines were analyzed using the BioGPS database (http://biogps.org)^18^. BioGPS is a database for retrieving and organizing gene annotation resources. It provides gene expression data for cells or tissues obtained by microarray analysis. Using GeneAtlas U133 A, gcrma dataset, the target-organ location network was constructed.

### Studying the prognostic value of FDX1 for human cancers by using two databases

We examined the prognostic significance of FDX1 expression in human tumours by using the GEPIA and Kaplan–Meier Plotter (http://kmplot.com/analysis/) database^19,20^. On the basis of the GEPIA database, we explored the relationship between FDX1 expression and disease-free survival (DFS) and overall survival (OS) in 33 cancer types. The GEPIA database categorized groups based on median FDX1 expression. The Kaplan–Meier Plotter database calculates an optimal cutoff value to classify groups automatically. In 21 cancer types, we identified associations between FDX1 expression and OS and relapse-free survival (RFS) using the Kaplan–Meier Plotter database. Hazard ratios (HRs) with corresponding log-rank *P*-values and 95% confidence intervals (CIs) were calculated. Statistical significance was determined by a *P*-value of less than 0.05.

### External validation will be performed using the PrognoScan database

To evaluate the result of prognostic value of FDX1 by using the GEPIA and Kaplan–Meier Plotter database, we used the PrognoScan database (http://www.abren.net/PrognoScan/) for external evaluation. In this database, gene transcription and survival time are plotted according to individual datasets based on publicly available cancer datasets^21^. The threshold values were corrected *P* = 0.05 and Cox *P* = 0.05.

### Analyzing FDX1 expression in molecular and immune subtypes of human cancers

Online integrated repository portal called TISIDB (http://cis.hku.hk/TISIDB/index.php) gathers data from TCGA, which contains extensive data sets related to human cancer^22^. In the TISIDB database, correlations were examined between expression of FDX1 and immunological or molecular subtypes of different cancers((C1 (wound healing); C2 (IFN-gamma dominant); C3 (inflammatory); C4 (lymphocyte depleted); C5 (immunologically quiet); C6 (TGF-βdominant)). Statistically significant differences were defined as *P*-value < 0.05.

### Analysis of the correlation between FDX1 expression and tumor mutational burden (TMB) and microsatellite instability (MSI) in human cancers

The TMB and MSI scores were obtained from TCGA. Spearman's correlation method was used to analyze the relationship between the expression of FDX1 and TMB or MSI. As shown in the figure, the horizontal axis shows the correlation coefficient between FDX1 and MSI or TMB, whereas the ordinate represents different type of cancer. A larger dot indicates a larger correlation coefficient, and different colors indicate a significant *P*-value.

### Analyzing the correlation between FDX1 expression and immune infiltration by using TIMER2.0

In order to analyze the correlation between FDX1 expression and immune infiltrates across all TCGA tumors, TIMER2.0 tool was used^17^. Cancer-associated fibroblasts, monocyte, endothelial cell and T cell follicular helper were selected for detailed analysis. To perform estimations, the following algorithms were used: EPIC, CIBERSORT, CIBERSORT-ABS, QUANTISEQ, MCPCOUNTER, XCELL, TIDE and TIMER. These algorithms have been thoroughly evaluated^23^, each having unique properties and strengths. For example, different tissue types may elicit distinct cancer-cell intrinsic expression and result in varying immune contexts^24^. TIMER is the only method that considers tissue specificity when estimating immune cell populations, though it only estimates for six immune cell types. XCELL is capable of estimating a higher number of different immune cell types, but may not detect signals from homogeneous samples. CIBERSORT can deconvolve more detailed T-cell subsets. EPIC has the advantage of directly producing scores that can be interpreted as cell fractions.

### FDX1 expression level and immune checkpoint (ICP) genes in pan-cancer

A series of molecules called ICP are expressed on immune cells, and they regulate the immune response by maintaining optimal immune function in the body. Researchers found that ICP genes play a significant role in immune cell infiltration and immunotherapy^25^. To explore the potential of FDX1 in immunotherapy, we investigated the association between FDX1 expression and ICP genes in human cancers. An analysis of the relationship between FDX1 expression and 47 common immune checkpoint genes was conducted. In heat maps, the horizontal axis represents cancer types, the vertical axis represents immune checkpoint genes, and the colors represent correlation coefficients. R software v 4.1.0 was used for statistical analysis (∗ *P* < 0.05, ∗∗ *P* < 0.01).

### FDX1 genomic alterations in human cancers explored by database

Currently, c-BioPortal (http://cbioportal.org) contains 225 cancer studies for interactive analysis of multidimensional cancer genomics datasets^26^. Thus, FDX1 genomic alterations in human cancers were investigated using the c-BioPortal database.

### Potentially regulatory pathways analysis

We obtained RNAseq data and related clinical information of pan-cancer from the TCGA database. We analyzed the correlation between FDX1 expression and the single-sample gene set enrichment analysis (ssGSEA) scores using potential regulatory pathways. The analysis was conducted using R version 4.1.0 and *P*-value < 0.05 were considered statistically significant.

### Drug sensitivity analysis

The data of cancer cell lines from the Genomics of Drug Sensitivity in Cancer (GDSC) was analyzed using the GSCA (http://bioinfo.life.hust.edu.cn/GSCA/#/drug) to determine the relationship between drug sensitivity and gene expression^27^. Pearson correlation was employed to assess the correlation between FDX1 expression and small molecule drug sensitivity, as represented by IC50 values. Using the TCGA database, the IC50 of each KIRC sample was predicted for clinically validated pharmacotherapeutic agents and statistically significant drugs based on the GDSC database. Differences in IC50 between low-FDX1 and high-FDX1 groups were compared using the Wilcoxon signed-rank test and were displayed in box plots generated by the R packages pRRophetic and ggplot2.

### Molecular docking simulations

We performed molecular docking simulations to further substantiate the effectiveness of chemotherapeutic agents and their potential interactions with FDX1. The 3D structure of FDX1 protein (PDB: 3p1m) was obtained from the PDB database (http://www.rcsb.org/pdb/home/home.do), followed by the removal of water molecules and ligands from the active site. The top three chemotherapeutic agents Elesclomol, Sorafenib and Temsirolimus were downloaded as small molecule structures from PubChem Compound (https://pubchem.ncbi.nlm.nih.gov/) the zinc15 database (https://zinc.docking.org/).

AutoDockTools 1.5.6 was utilized to process receptor proteins and small molecule ligands, including adding polar hydrogens, calculating charges, and defining rotatable bonds. The receptor protein docking site parameters were adjusted to encompass the active pocket region for small molecule ligand binding. The grid box was centered at (1.081, 0.891, 0.922) Å, and grid point distance was 0.05 nm. Subsequently, Molecular docking studies were performed by Autodock Vina 1.2.2 (http://autodock.scripps.edu/).

### Studying the expression of FDX1 in different clinical subgroups of KIRC

RNA-seq and clinical data on 31 cancer types are gathered for the UALCAN (http://ualcan.path.uab.edu) database through the TCGA^28^. Databases like this one can be used to analyze gene expression in tumors and normal tissues. Individual gene expression and clinicopathological characteristics of cancers were analyzed using the method.

### Exploration of FDX1 coexpression networks using a database

LinkedOmics (http://www.linkedomics.org/login.php) is a visual tool for exploring gene expression profiles^29^. Based on Pearson's correlation coefficient, LinkedOmics was used to determine FDX1 coexpression genes and the results were visualized using volcano plots and heat maps. Next, we examined the Gene Ontology biological process (GO_BP), as well as KEGG pathways of FDX1.

## Results

### In human cancers, FDX1 is differentially expressed between tumors and normal tissues

**Figure 1:**
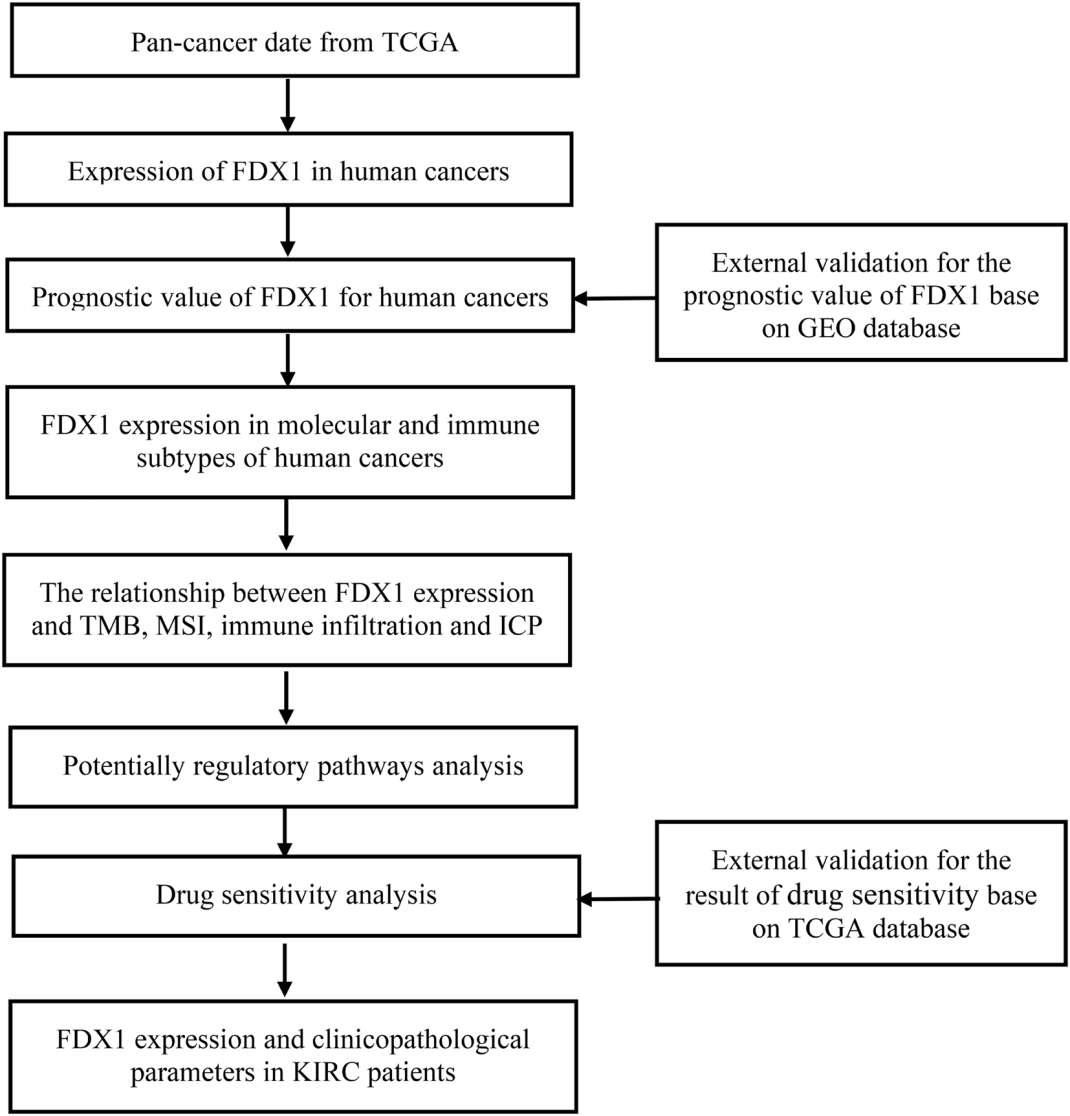
The flow sheet of this study.

**Figure 2:**
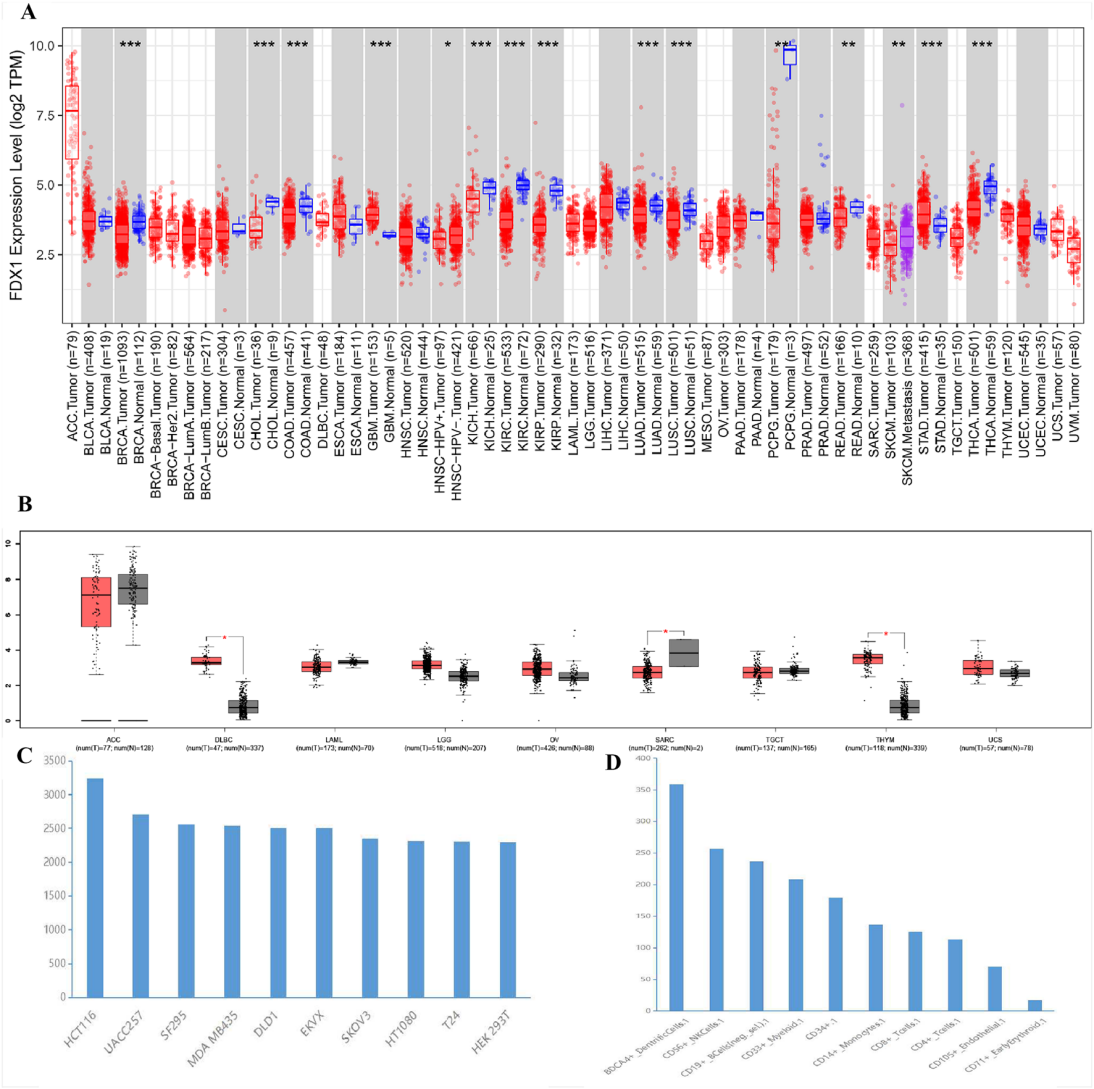
FDX1 expression levels in human cancers. (**A**) using TIMER2 to analyze the expression of FDX1 in different cancers or specific cancer subtypes. (*P < 0.05, **P < 0.01, ***P < 0.001). (**B**) FDX1 expression in several cancers and paired normal tissue in the GEPIA database. (**C**) the expression of FDX1 in different cancer cell lines analyzed by the BioGPS database. (**D**) the expression of FDX1 in normal tissue analyzed by the BioGPS database.

The flow sheets in this study have been shown in Fig. 1. Based on the results of the TIMER2.0 database, FDX1 expression was significantly lower in BRCA (breast invasive carcinoma), COAD (colon adenocarcinoma), CHOL(cholangiocarcinoma), HNSC-HPV (head and neck cancer), KIRP (kidney renal papillary carcinoma), KICH (kidney chromophobe), KIRC, LUAD (lung adenocarcinoma), LUSC (lung squamous cell carcinoma), PCPG (pheochromocytoma and paraganglioma), PEAD (rectum adenocarcinoma), THCA (thyroid carcinoma) and SKCM (skin cutaneous melanoma) than in adjacent normal tissue. But, FDX1 mRNA expression was high only in GBM (glioblastoma multiforme) and STAD (stomach adenocarcinoma) (Fig. 2A). Results of the GEPIA analysis are shown as supplemental data for cancers without paired normal tissues in the TIMER2.0 database. Additionally, the results showed that FDX1 mRNA expression level was significantly higher in DLBC (lymphoid neoplasm diffuse large B-cell lymphoma) and THYM (thymoma) except SARC (sarcoma) (Fig. 2B). By using the BioGPS database, we investigated the expression of FDX1 in different normal tissues and cancer cell lines, and found that almost all cancer cell lines expressed FDX1. Figure 2C shows ten cancer cell lines expressing the highest level of FDX1. The highest expression of FDX1 was found in immune cells (Fig. 2D). According to the above results, FDX1 was expressed differently in different cancer tissues and might be involved with immune regulation.

### FDX1 is a prognostic biomarker for pan-cancer

**Figure 3:**
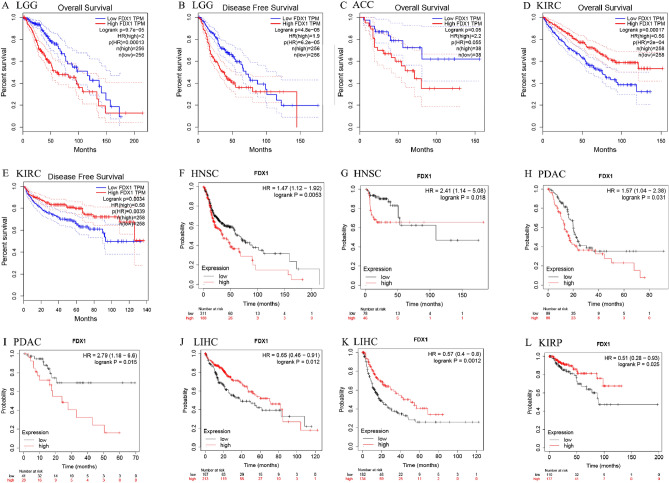
Analyzing the Kaplan–Meier survival curve of human cancers with high and low FDX1 expression using the GEPAI database (**A**–**D**) and the Kaplan–Meier plotter database (**E**–**L**).

**Figure 4:**
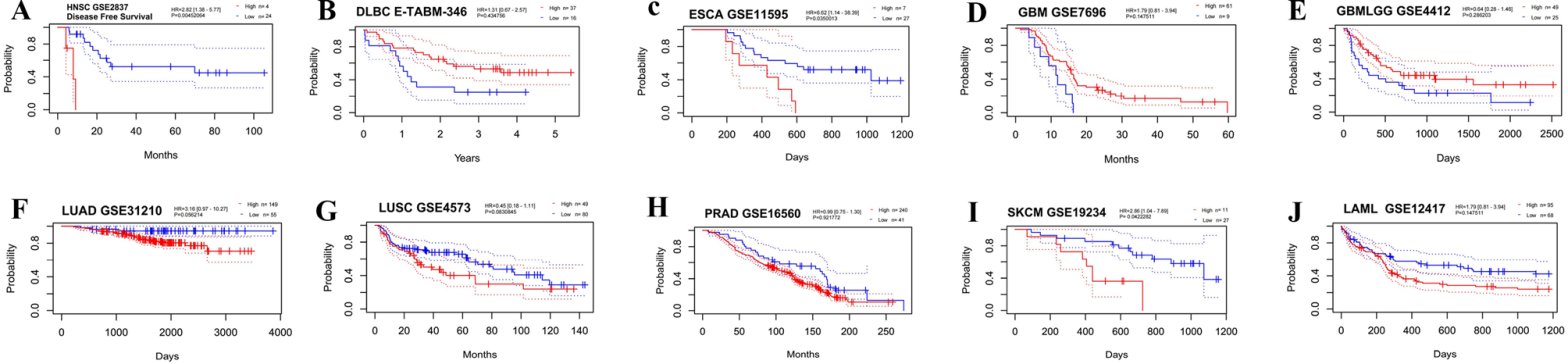
External validation for the prognostic value of FDX1 by using the PrognoScan database. (**A**) in HNSC, (**B**) in DLBC, (**C**) in ESCA, (**D**) in GBM, (**E**) in GBMLGG, (**F**) in LUAD, (**G**) in LUSC, (**H**) in PRAD, (**I**) in SKCM, (**J**) in LAML.

Several databases examined FDX1 expression in human cancers to determine its prognostic value. According to the result of GEPIA, it show that higher FDX1 expression was associated with poorer disease-free survival (DFS) and overall survival (OS) in LGG (brain lower grade glioma) (n = 256, DFS: HR = 2.0, *P* = 0.000097; n = 256, OS: HR = 1.9, *P* = 0.000048; Fig. 3A,B). Additionally, patients with higher FDX1 expression had poorer OS in ACC (adrenocortical carcinoma) (n = 38, HR = 2.2, *P* = 0.05; Fig. 3C). However, higher FDX1 expression may related to better DFS and OS in KIRC (n = 258, DFS: HR = 0.58, *P* = 0.0034; n = 258, OS: HR = 0.56, *P* = 0.00017; Fig. 3D,E). In the Kaplan–Meier plotter database, higher FDX1 expression was associated with poorer OS and relapse-free survival (RFS) in HNSC (head and neck squamous cell carcinoma) (n = 499, OS: HR = 1.47, *P* = 0.0053; n = 124, RFS: HR = 2.41, *P* = 0.018; Fig. 3F,G) and PDAC (pancreatic ductal adenocarcinoma) (n = 177, OS: HR = 1.57, *P* = 0.031; n = 69,RFS: HR = 2.79, *P* = 0.015; Fig. 3H,I). However, patients with higher FDX1 expression was associated with better OS and RFS in LIHC (liver hepatocellular carcinoma) (n = 370, OS: HR = 0.65, *P* = 0.012; RFS:HR = 0.57, *P* = 0.0012; Fig. 3J,K). In addition, patients with higher FDX1 expression was associated with better OS in KIRP (n = 287, OS: HR = 0.51, *P* = 0.012, Fig. 3L). In Supplementary Fig. 1, as analyzed by Kaplan–Meier plotters database, the FDX1 and OS or RFS correlation is shown. Finally, one independent DLBC dataset E-TABM-346 and nine GEO datasets, consisting of GSE19234, GSE16560, GSE4573, GSE31210, GSE31210, GSE2837, GSE4412, GSE7696 and GSE11595, were introduced to further confirm the predictive value of FDX1 in prognosis of pan-cancer. The results presented that patients with high expression of FDX1 had poor OS or DFS in HNSC, ESCA and SKCM (Fig. 4). In general, the results in both database are similar. We noted that the validation result for SKCM was not consistent with that of the TCGA dataset, this may result from the different sample size. According to the above results, FDX1 expression was closely associated with the prognosis of various cancer types.

### Immune and molecular subtypes of human cancer are related to the expression of FDX1

**Figure 5:**
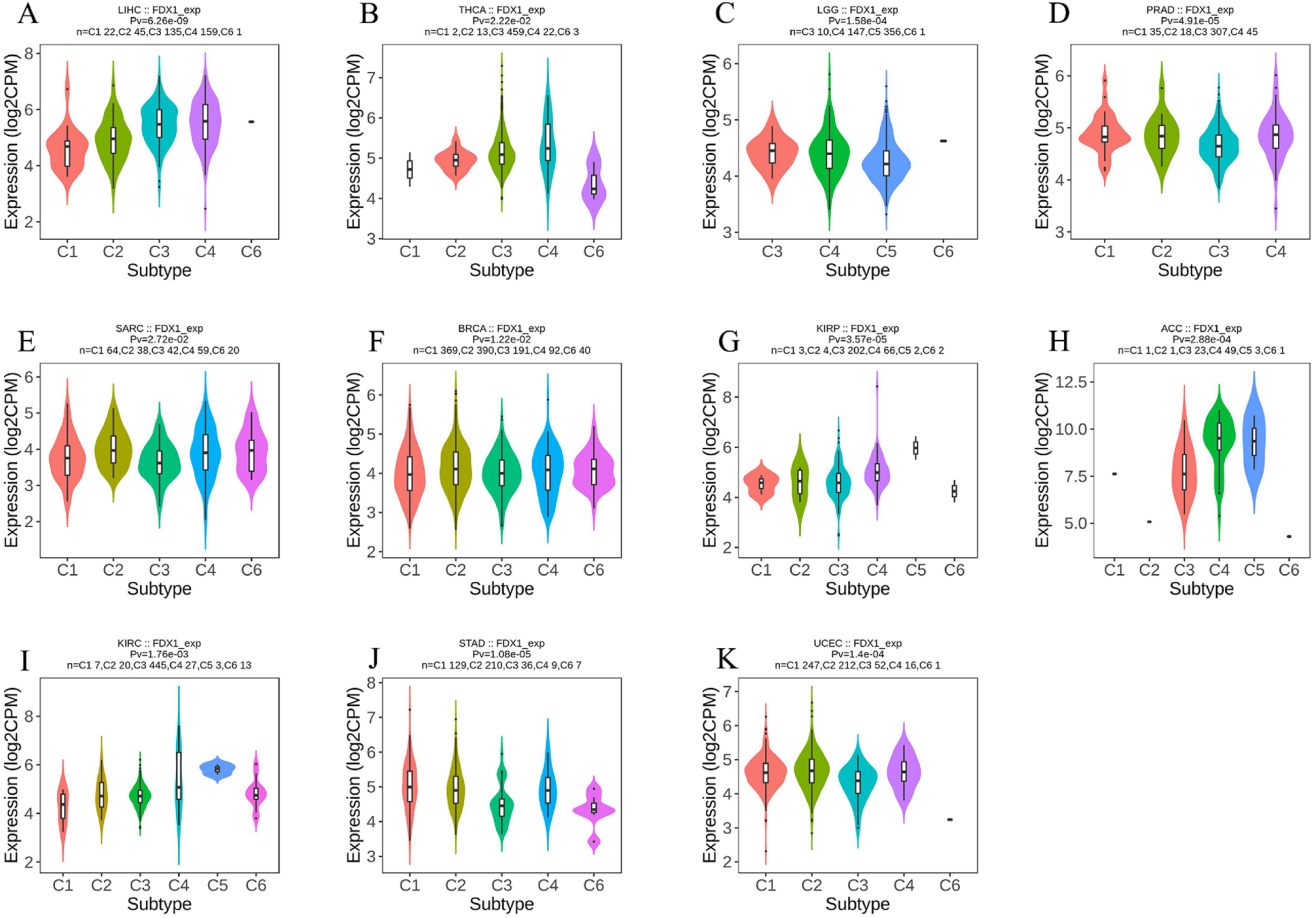
An analysis of the relationship between FDX1 expression and pan-cancer immune subtypes. (**A**) in LIHC, (**B**) in THCA, (**C**) in LGG, (**D**) in PRAD, (**E**) in SARC, (**F**) in BRCA, (**G**) in KIRP, (**H**) in ACC, (**I**) in KIRC, (**J**) in STAD, (**K**) in UCEC.

**Figure 6:**
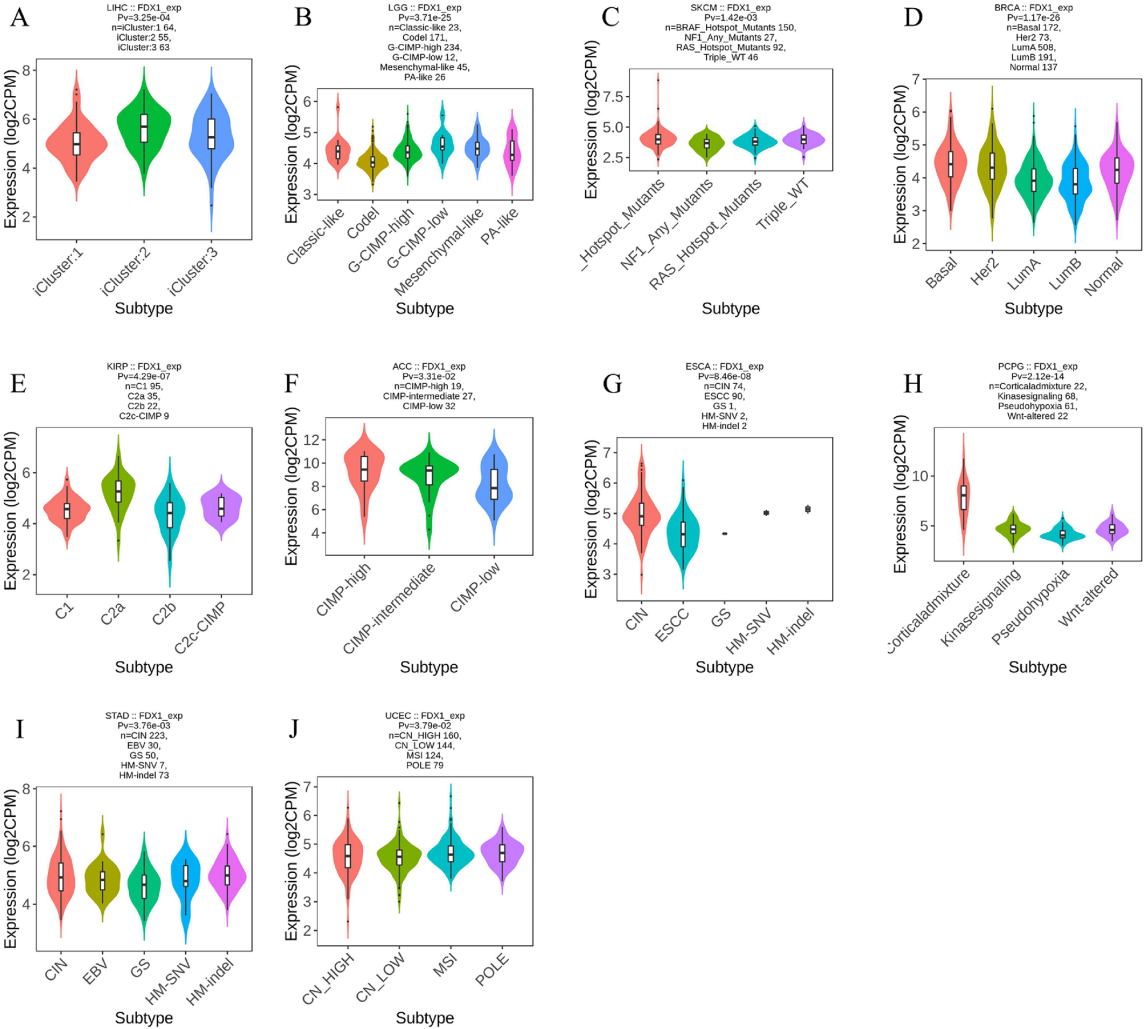
An analysis of the relationship between FDX1 expression and pan-cancer molecular subtypes. (**A**) in LIHC, (**B**) in LGG, (**C**) in SKCM, (**D**) in BRCA, (**E**) in KIRP, (**F**) in ACC, (**G**) in ESCA, (**H**) in PCPG, (**I**) in STAD, (**J**) in UCEC.

By using the TISIDB website, we investigated the role of FDX1 expression on immune and molecular subtypes in human cancers. The results showed that FDX1 expression may be associated with specific different immune subtypes in ACC, BRCA, KIRP, KIRC, LIHC, LGG, PRAD (prostate adenocarcinoma), SARC, STAD, THCA and UCEC (uterine corpus endometrial carcinoma) (Fig. 5). Additionally, FDX1 expression varied among the immune subtypes of a given cancer type. As an example of LIHC, FDX1 showed low expression in C1 types and high expression in C3 and C4 types. Different molecular subtypes of cancer were significantly associated with FDX1 expression in ACC, BRCA, KIRP, ESCA, LIHC, PCPG, LGG, SKCM, STAD and UCEC (Fig. 6). As a result of the above findings, we concluded that FDX1 expression varies between molecular subtypes and immune subtypes of different cancer types in humans.

### Analyzing the correlation between FDX1 expression and immune cell infiltration using TIMER2 tool

**Figure 7:**
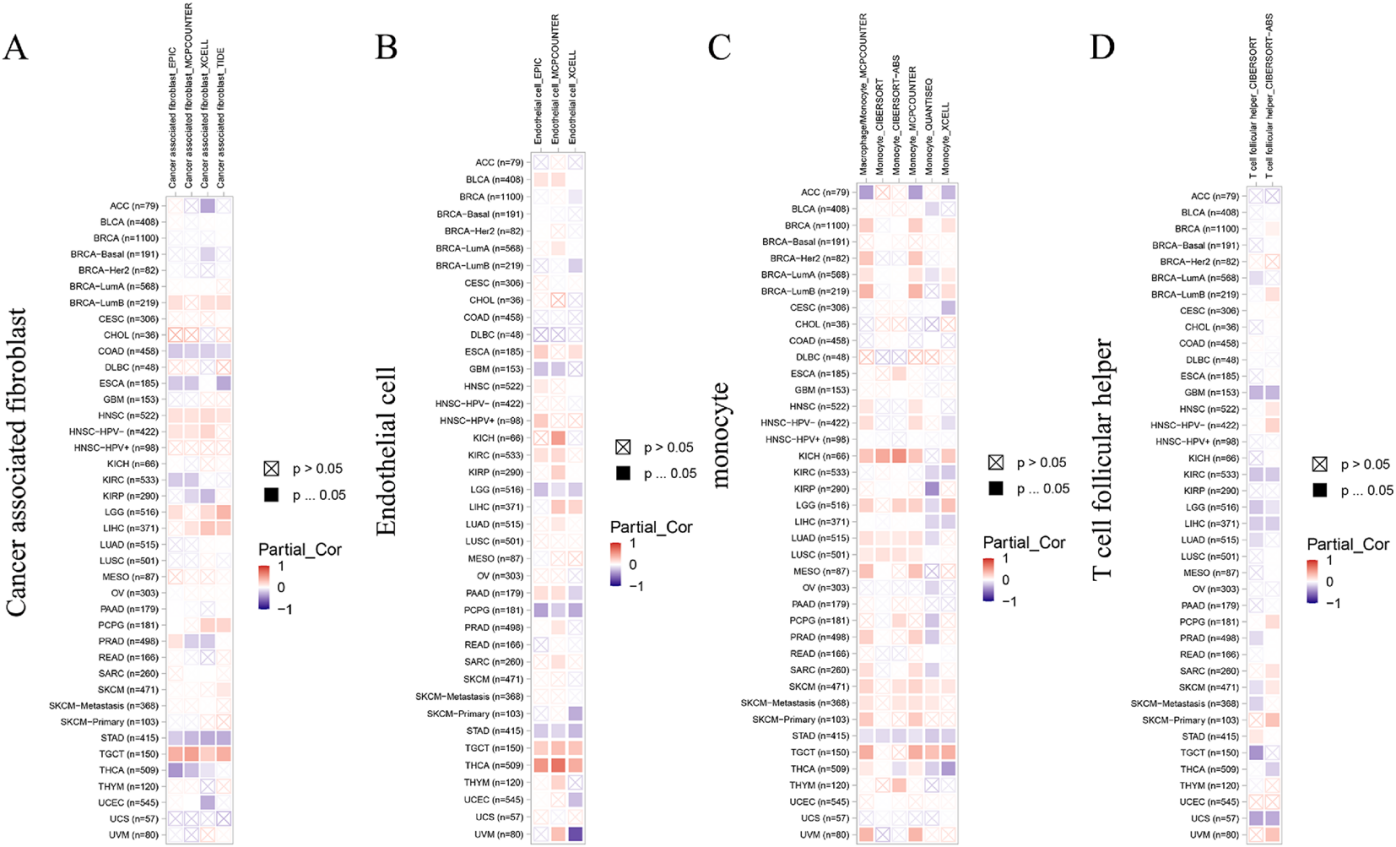
In TIMER 2.0, different algorithms are used to determine the association between immune infiltration and FDX1.

Following our demonstration of differential FDX1 expression in different immune subtypes, an analysis of FDX1 and immune cell infiltration in human cancer was carried out. Hence, we investigated the correlation between the infiltration level of different endothelial and immune cells and FDX1 expression in multiple tumor types from TCGA using the CIBERSORT, CIBERSORT-ABS, TIMER, TIDE, XCELL, QUANTISEQ, MCPCOUNTER and EPIC algorithms. It is interesting to note that we found FDX1 expression was negatively correlated with the estimated infiltration of cancer-associated fibroblasts and monocytes for the STAD. For LGG, PCPG, and STAD, FDX1 expression and endothelial cell infiltration also showed negative correlations, while a positive correlation was found for TGCT and THCA tumors. In addition, the relation between T cell follicular helper and FDX1 expression was showed negative correlation in GBM, KIRC, LGG, LIHC and UCS (Fig. 7).

### FDX1 expression is associated with immune checkpoint (ICP) genes in human cancers

**Figure 8:**
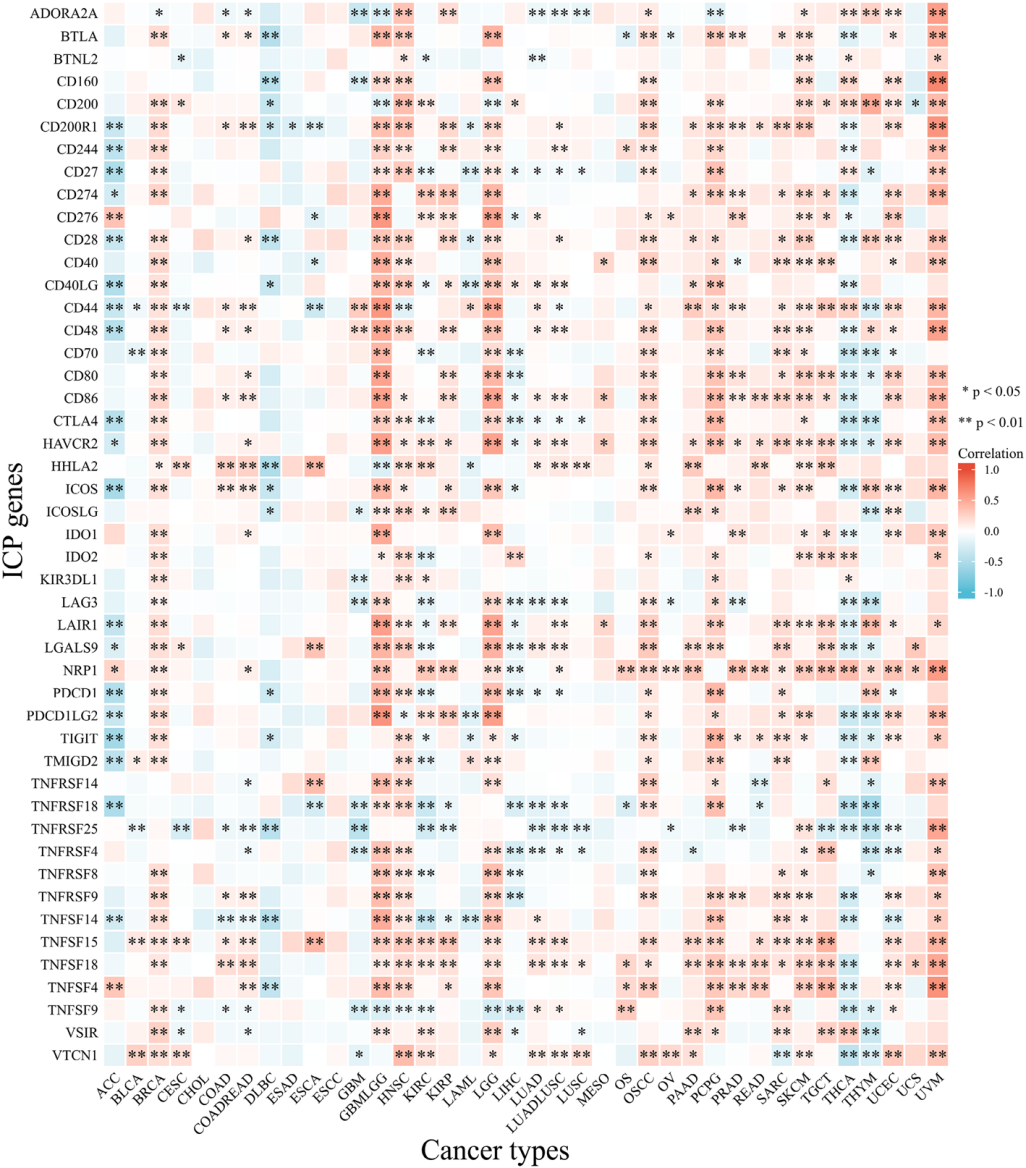
The relationship between FDX1 expression and pan-cancer immune checkpoint genes. *P < 0.05; **P < 0.01.

We found strong relationships between 47 ICP genes and FDX1 expression in several cancer types, except CHOL, ESAD, ESCC, MESO and UCS (Fig. 8). In BRCA, GBMLGG (glioma), HNSC, LGG, OSCC (oral squamous cell carcinoma), PAAD (pancreatic adenocarcinoma), PCPG, PRAD, READ, SARC, SKCM, TGCT (testicular germ cell tumors), UCEC and UVM (uveal Melanoma), There was a positive relationship between FDX1 expression and immune checkpoint genes especially in OSCC, where 37 of 47 immune checkpoint genes displayed a relationship with FDX1. These findings suggest that FDX1 may be involved in coordinating the activity of these ICP genes through different signal transduction pathways, making it an attractive target for immunotherapy. In immunotherapies targeting ICP genes, high FDX1 expression might predict good therapeutic results. There is a negative relationship between FDX1 expression and ICP genes in ACC, DLBC, LAML (acute myeloid leukemia), LIHC, THCA and THYM, which suggests that high FDX1 expression may predict unsatisfactory immunotherapy results when targeting these genes. In contrast, FDX1 inhibitors may provide an alternative treatment option. Potentially serving as a pan-cancer biomarker or a new immunotherapy target, FDX1 may have a significant impact on predicting immunotherapy response or helpful in achieving promising therapeutic outcomes.

### FDX1 expression is associated with tumor mutational burden (TMB) and microsatellite instability (MSI)

**Figure 9:**
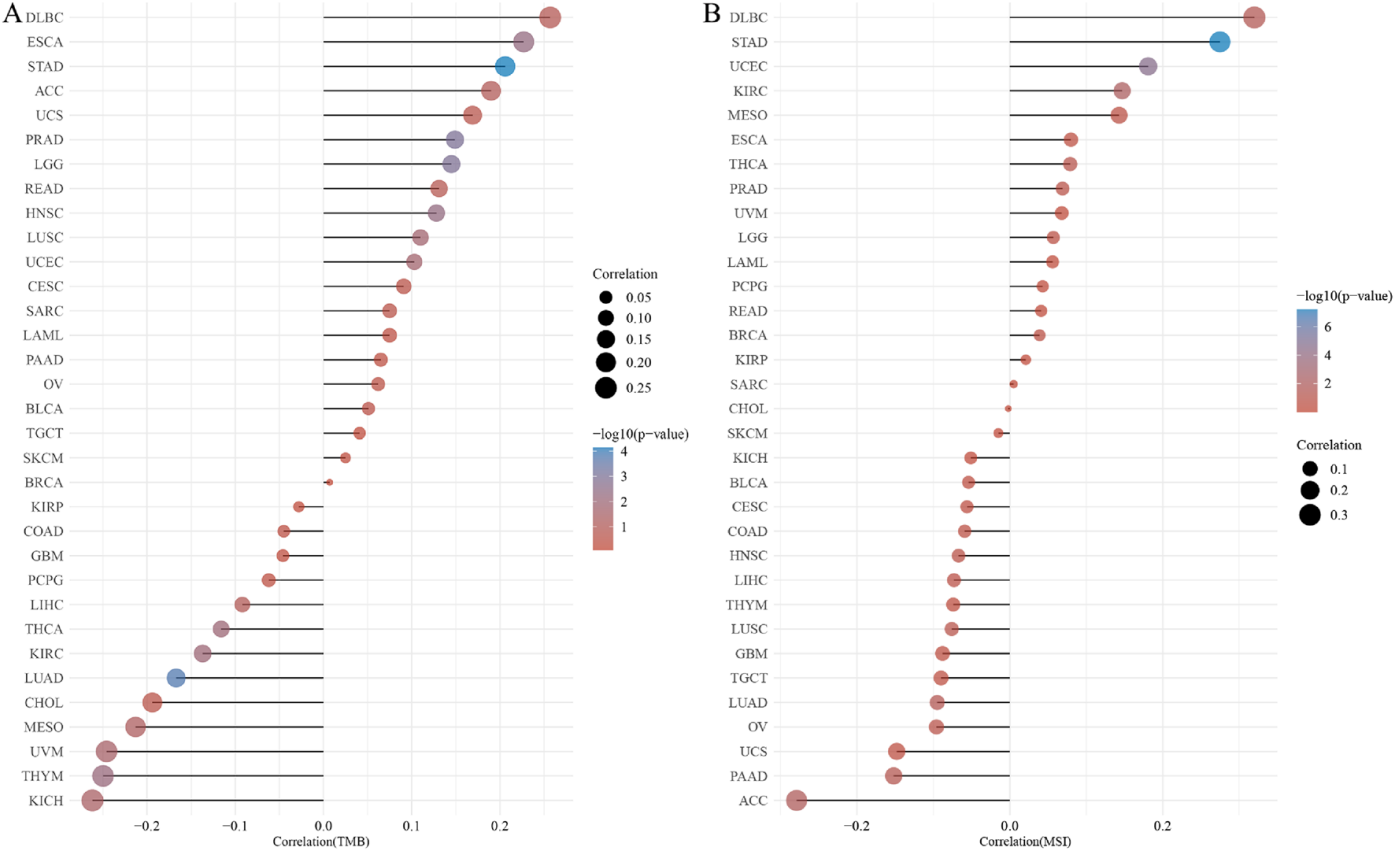
The relationship between FDX1 expression and TMB (**A**) and MSI (**B**) in human cancers.

FDX1 was investigated for its role in the immune mechanism and immune response of the tumor microenvironment (TME) by examining its relationship to tumor mutational burden (TMB) and Microsatellite Instability (MSI). The level of TMB and MSI in tumor microenvironment is associated with antitumor immunity and may predict the effectiveness of immunotherapy^30,31^. Our results showed that FDX1 expression had significant positive associations with TMB in UCEC, LUSC, ESCA, HNSC, LGG, PRAD and STAD and negative relations in KICH, UVM, THCA, KIRC, THYM and LUAD (Fig. 9A). For MSI, there were positive correlations with FDX1 expression in DLBC, KIRC, UCEC and STAD and negative correlations with in PAAD, LUAD and ACC (Fig. 9B). As a result, the above finding further strengthened our hypothesis that FDX1 may regulate the immune mechanism and composition within TME and influence antitumor immunity.

### Pathway correlation analysis of molecular

**Figure 10:**
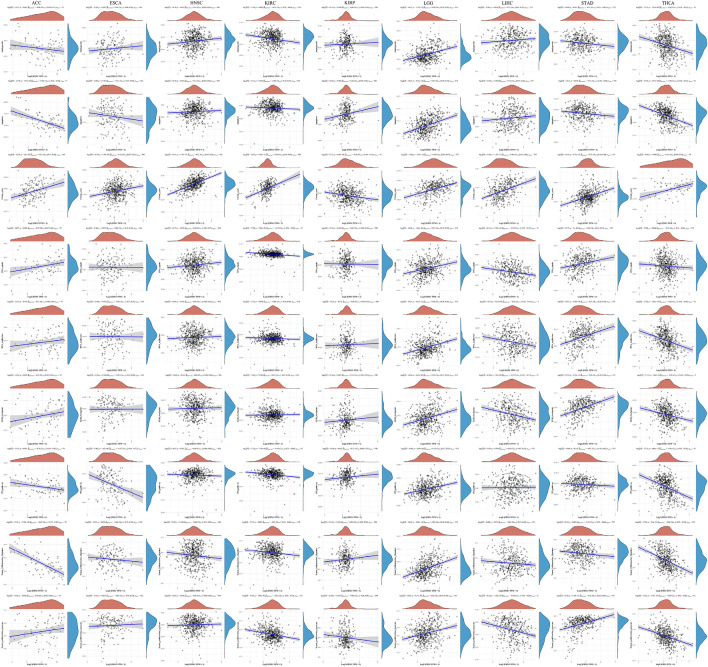
Pathway correlation analysis of FDX1.

We separately assessed some common functional pathways by ssGSEA algorithm, including: angiogenesis, apoptosis, citrate_cycle, DNA_repair, DNA_replication, G2M_checkpoint, P53_pathway, tumor_inflammation and tumor_proliferation. The results suggest that FDX1 was positively correlated with citrate_cycle, DNA_repair, G2M_checkpoint and tumor_proliferation, while negatively correlated with apoptosis, P53_pathway and tumor_inflammation in ACC. For ESCA, FDX1 was positively correlated with citrate_cycle, while negatively correlated with P53_pathway. For HNSC, FDX1 was positively correlated with angiogenesis, apoptosis and citrate_cycle. For KIRC, FDX1 was positively correlated with citrate_cycle, while negatively correlated with angiogenesis, DNA_repair, P53_pathway, tumor_inflammation and tumor_proliferation. For KIRP, FDX1 was positively correlated with apoptosis and P53_pathway, while negatively correlated with citrate_cycle. For LGG, FDX1 was positively correlated with angiogenesis, apoptosis, citrate_cycle, DNA_repair, DNA_replication, G2M_checkpoint, P53_pathway, tumor_inflammation and tumor_proliferation. For LIHC, FDX1 was positively correlated with angiogenesis, apoptosis and citrate_cycle, while negatively correlated with DNA_repair, DNA_replication, G2M_checkpoint and tumor_proliferation. For STAD, FDX1 was positively correlated with citrate_cycle, DNA_repair, DNA_replication, G2M_checkpoint and tumor_proliferation, while negatively correlated with angiogenesis, apoptosis and tumor_inflammation. For THCA, FDX1 was positively correlated with citrate_cycle, while negatively correlated with angiogenesis, apoptosis, DNA_repair, DNA_replication, G2M_checkpoint, P53_pathway, tumor_inflammation and tumor_proliferation (Fig. 10).

### Drug sensitivity analysis

**Figure 11:**
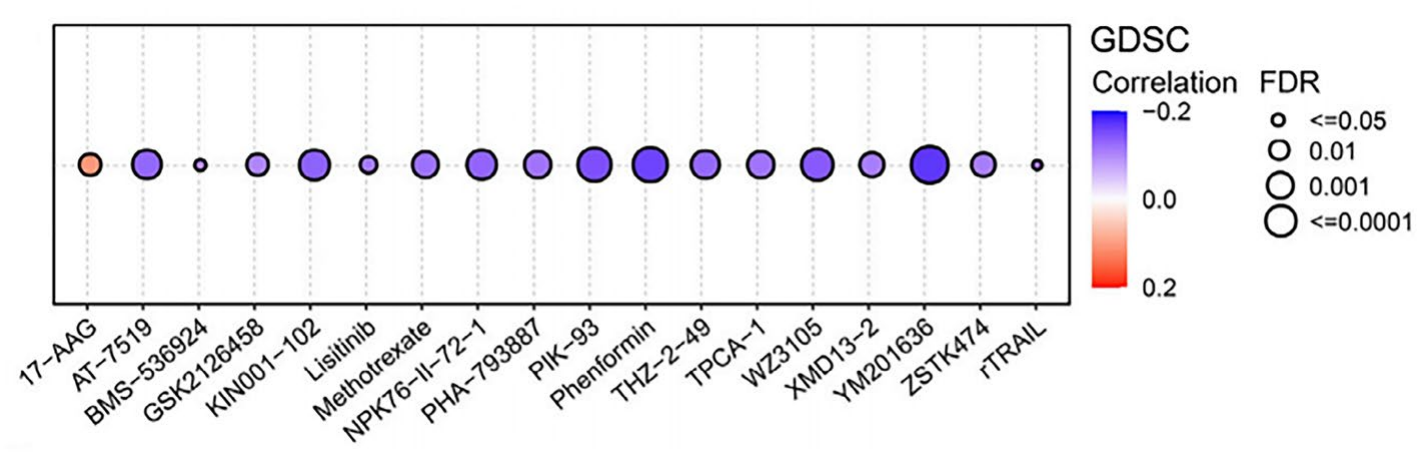
Predictive drugs based on the FDX1 expression in pan-cancer from the GDSC database.

**Figure 12:**
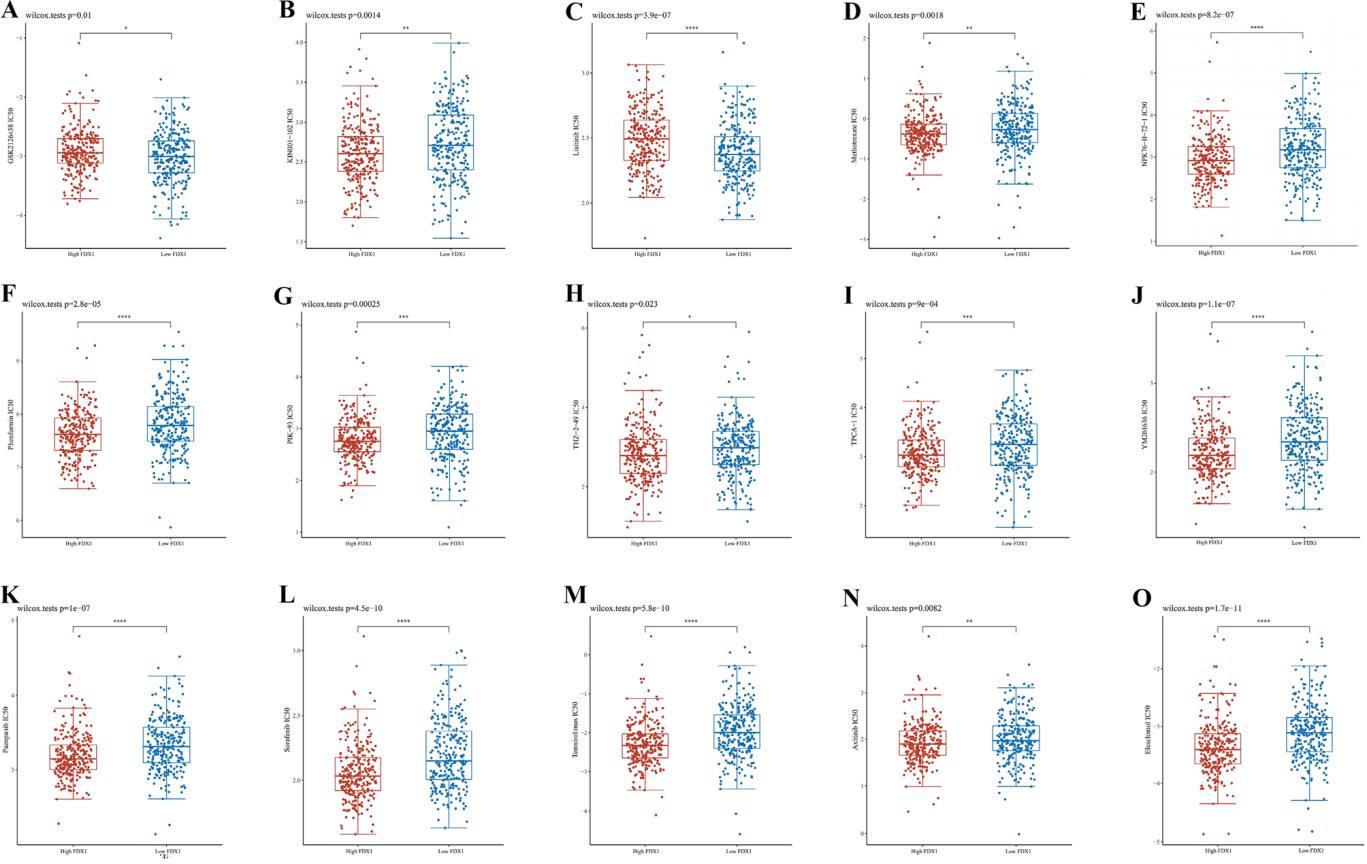
Sensitivity analysis for medical treatment in FDX1 high expression groups and low expression groups of KIRC patients. (**A**) in GSK2126458, (**B**) in KIN001-102, (**C**) in Lisitinib, (**D**) in Methotrexate, (**E**) in NPK76-II-72-1, (**F**) in Phenformin, (**G**) in PIK-93, (**H**) in THZ-2-49, (**I**) in TPCA-1, (**J**) in YM201636, (**K**) in Pazopanib, (**L**) in Sorafenib, (**M**) in Temsirolimus, (**N**) in Axitinib, (**O**) in Elesclomol.

**Figure 13:**
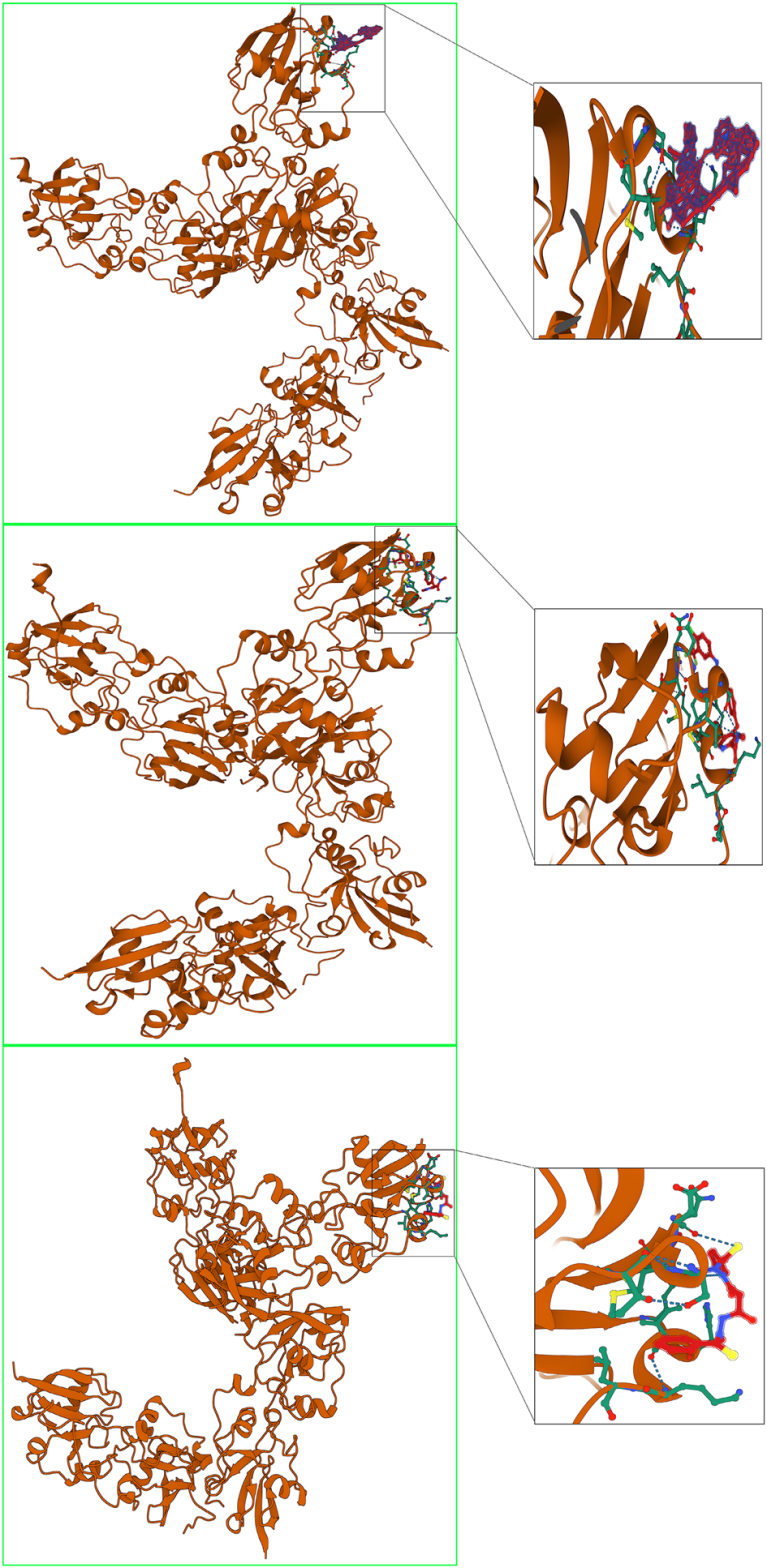
Molecular docking results of protein FDX1 (3p1m) with Temsirolimus, Sorafenib and Elesclomol.

As shown in Fig. 11, FDX1 expression was negatively correlated with sensitivity to drugs such as PIK-93, phenformin and YM201636 and positively correlated with sensitivity to 17-AAG. Further validation of these drug was made in KIRC sample in the TCGA database. The difference in mean IC50 between the high and low FDX1-expressing groups was statistically significant in fifteen drugs (BMS-536924 could not be evaluated because of overlapping study samples). GSK2126458 and lisitinib were predicted to have better therapeutic effect on low FDX1-expressing subgroup, as shown by the lower IC50 value. However, KIN001-102, Methotrexate, NPK76-II-72-1, Phenformin, PIK-93, THZ-2-49, TPCA-1, YM201636, Pazopanib, Sorafenib, Temsirolimus, Axitinib and Elesclomol were predicted to have better therapeutic effect on high FDX1-expressing subgroup. (Fig. 12) Considering the strong correlation between chemotherapeutic drug sensitivity and FDX1, we employed molecular docking techniques to further assess the potential targeting effects of these chemotherapeutic agents on FDX1. Notably, the binding free energy of FDX1 and Temsirolimus (A), Sorafenib (B) and Elesclomol (C) are − 10.263 kcal/mol, − 5.901 kcal/mol and − 5.435 kcal/mol, respectively (Fig. 13). These findings indicate that multiple chemotherapeutic drugs demonstrate outstanding binding affinity to FDX1.

### FDX1 gene and expression altered in human cancers

**Figure 14:**
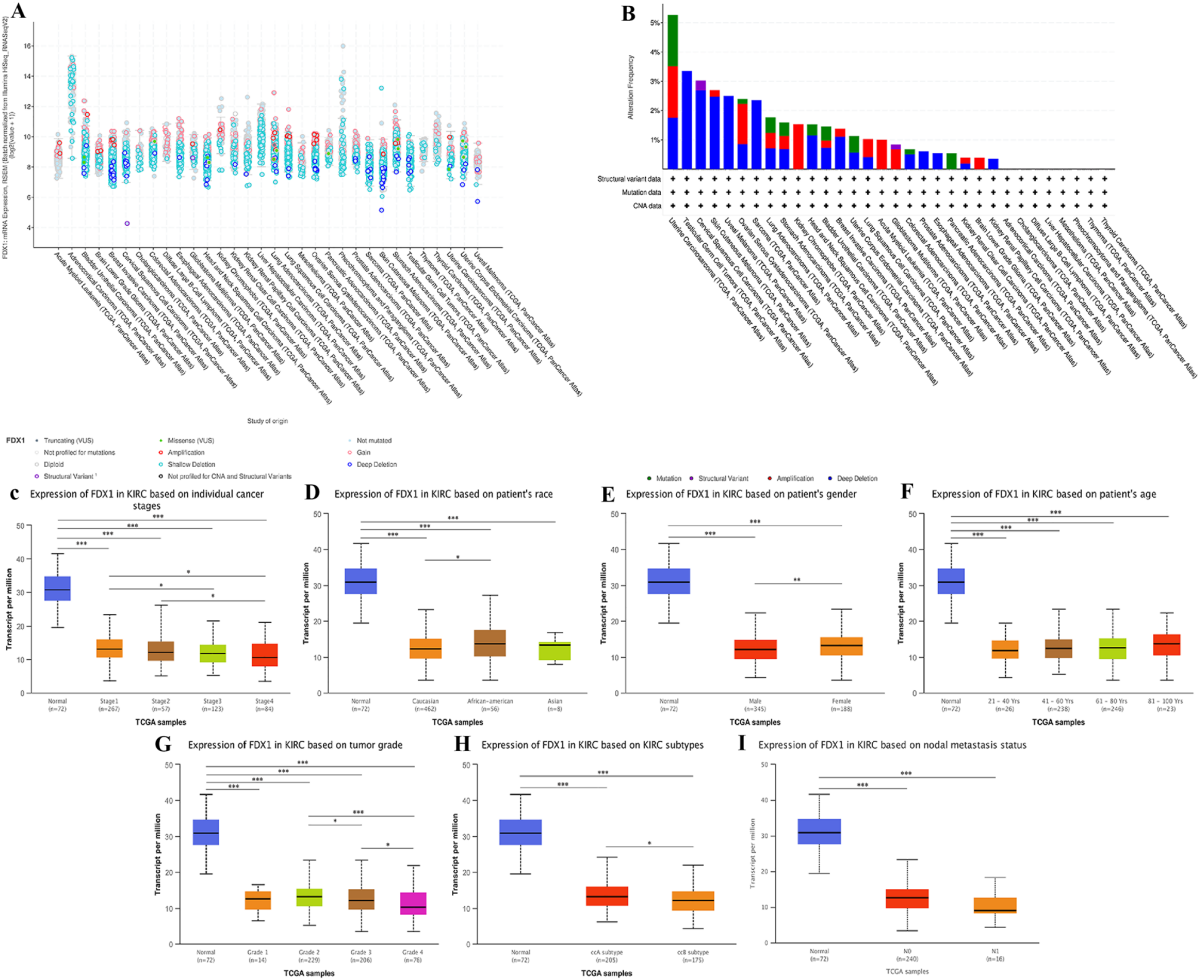
FDX1 genomic alterations in pan-cancer analyzed by the cBioPortal database (**A**–**B**) and FDX1 differential expression in KIRC with different clinical subgroups analyzed by the UALCAN database(C–I). (*P < 0.05, **P < 0.01, ***P < 0.001).

On the cBioPortal website, FDX1 genomic alterations were analyzed in human cancers. Genomic alterations of FDX1 were observed in 1.2% of patients. Diverse alterations in the FDX1 gene affected gene expression (Fig. 14A). UCS, TGCT, and CSCC showed the highest levels of copy number variation, while ACC, CHOL, DLBCL, LIHC, Mesothelioma, PCPG, Thymoma and THCA did not have any CNV at all (Fig. 14B). Next, we explored FDX1 expression in KIRC with different clinical features using the UALCAN database. A significant difference in FDX1 expression occurred in different cancer stages, patient race, patient sex, patient age, KIRC subtypes, tumor grade and nodal metastasis status of KIRC (Fig. 14C–I). Based on the results presented above, it appears that FDX1 genomic changes and differential expression are indeed found in cancer tissue, and possibly play a role in cancer progression and onset.

### Relationships between FDX1 expression and clinicopathological parameters in KIRC patients

**Figure 15:**
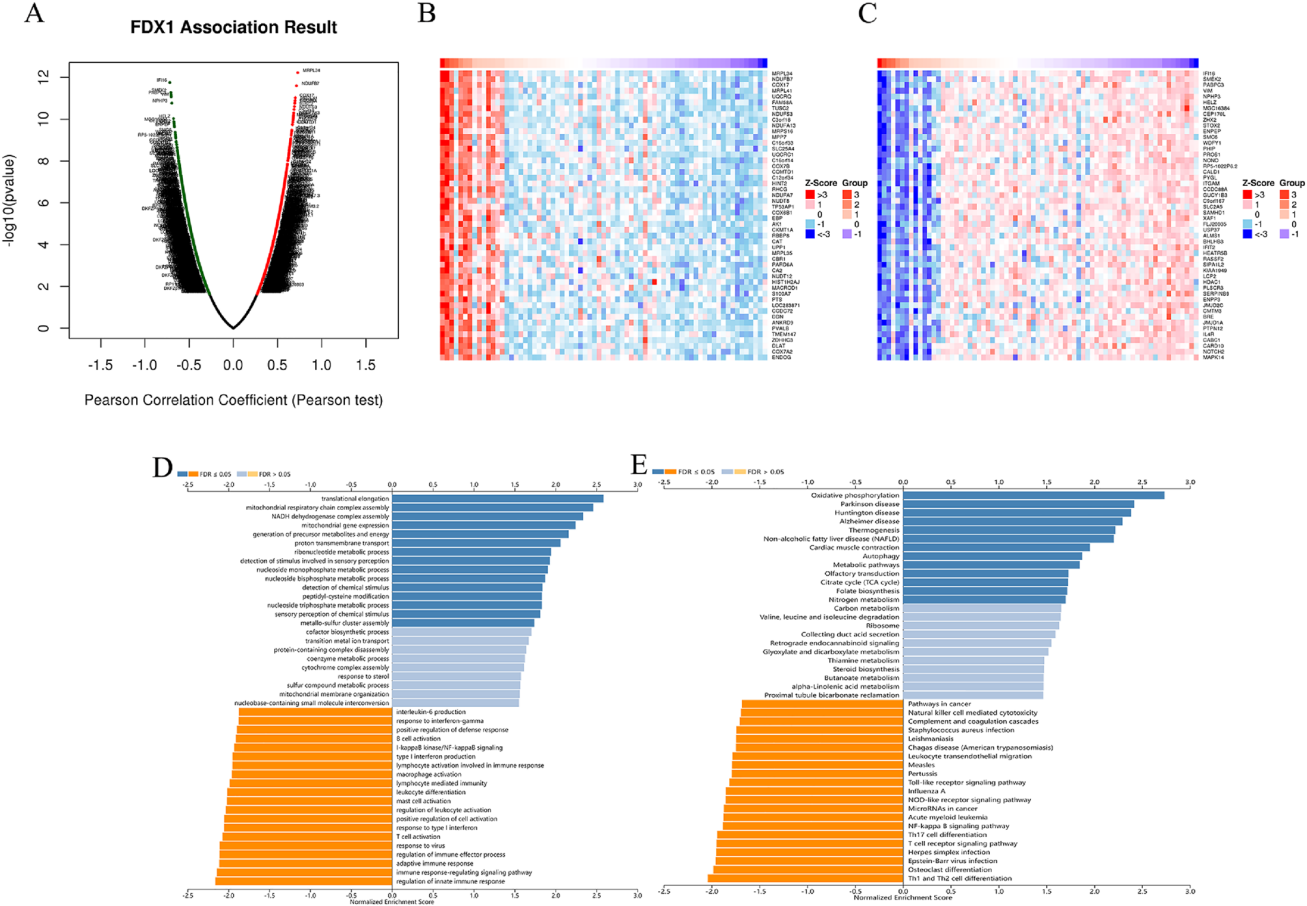
Function and pathway enrichment analyses for genes co-expressed with FDX1 in KIRC. (**A**) Highly correlated genes of FDX1 tested by Pearson test in KIRC cohort; Top 50 positive coexpression genes (**B**) and negative coexpression genes (**C**) of FDX1 in heat map in KIRC; (**D**, **E**) Genes of FDX1 co-expressed enrichment analysis performed by LinkedOmics.

According to the results above, FDX1 significantly associated with cancer immunity and prognosis. Following that, we investigated FDX1 coexpression networks using the LinkedOmics database, which validated its function in tumor tissue, and illustrated that possibility using KIRC. As is plotted in Fig. 15A, it showed that 1405 genes (dark red dots) correlated positively with FDX1, while 1827 genes (dark green dots) correlated negatively. The heat maps in Fig. 15B,C show the top 50 genes that are positively and negatively associated with FDX1, respectively. GO term annotation showed that co-expressed genes of FDX1 join mainly in translational elongation, mitochondrial respiratory chain complex assembly, NADH dehydrogenase complex assembly, mitochondrial gene expression and generation of precursor metabolites and energy, etc. (Fig. 15D). KEGG pathway analysis indicated enrichment in Oxidative phosphorylation, Parkinson disease, Huntington disease, Alzheimer disease and Thermogenesis, etc. (Fig. 15E). Results demonstrate that the expression network of FDX1 impacts prognosis and immune activation in KIRC.

## Discussion

A recent study indicated that copper ionophore treatment have potentially great therapeutic value in tumors^1,3^. FDX1 is known as an important regulatory factor in cuproptosis, however, its roles in tumor pathogenesis and immune infiltration remain unclear. Therefore, we performed a pan-cancer analysis of FDX1 using multiple databases to evaluate the features of gene expression, prognosis, and tumor immunity.

We used the GEPIA, TIMER and BioGPS databases to analyze the levels of FDX1 expression in cancers and normal tissues as a first step in our study. In line with previous studies, the expression of FDX1 was significantly lower in most cancer types, except GBM, STAD, DLBC and THYM^10–14^. According to these results, FDX1 may play a role in oncogenesis and the progression of tumors.

We then looked at the relationship between FDX1 expression and prognosis. In our study, high FDX1 expression patients had a worse prognosis in LGG, ACC, HNSC and PDAC. However, higher FDX1 expression meant better prognosis in KIRC and LIHC. Similar trends were found in the validation set. Notably, FDX1 showed a low expression in KIRC, and had a decreasing tendency with the development of cancer stages. The results presented above indicated that FDX1 could serve as a prognostic biomarker for pan-cancer. To determine its potential mechanism of action, we studied FDX1 expression in different immune subtypes and molecular subtypes of human cancers. According to our results, FDX1 expression differed significantly between molecular and immune subtypes of most cancers, which might indicate that FDX1 is a promising diagnostic biomarker for pan-cancers and that it regulates immunity as well. Moreover, FDX1 expression varied significantly in different clinical subgroups of KIRC, which suggests that FDX1 might be involved in the growth and progression of cancer.

TME is an area where tumor cells can evade immune surveillance. There is a significant influence of TME on clinical outcome and therapeutic response^32^. Research has identified a number of components involved in the formation of the TME, such as lymphocytes, cancer-associated fibroblasts, endothelial cells, the extracellular matrix, and mesenchymal stem cells^33–35^. The CIBERSORT, CIBERSORT-ABS, QUANTISEQ, XCELL, MCPCOUNTER, and EPIC algorithms were used to examine the correlation between the FDX1 gene and immune cells and stromal infiltration in this study. Our results showed that FDX1 expression had functions associated with cancer-associated fibroblasts, T cell follicular helper, monocyte and endothelial cells in different tumors. Within the TME, T cell follicular helper was found to exert tumor suppressive effects by promoting B cell maturation, affinity maturation, and antibody secretion^36–39^. Endothelial cells and Cancer-associated fibroblasts play pro-tumorigenic roles in the TME by secreting growth factors, cytokines, and chemokines, as well as by degrading extracellular matrix^40,41^. Tumor-associated macrophages and tumor-associated dendritic cells are formed from infiltrated monocytes, which affect the TME through diverse mechanisms. And it also induces angiogenesis, immune tolerance and tumor cell metastasis^42,43^. The association between FDX1 and TME might explain another reason for FDX1's prognostic significance in various cancers. According to several studies, FDX1 affects lung cancer prognosis by regulating fatty acid oxidation^10,44^. In summary, our study suggests that aberrant expression of FDX1 may be a significant factor contributing to TME.

We identified that the FDX1 coexpression network is involved in regulating immune response and presenting antigens. FDX1 and ICP genes were shown to be associated, providing a theoretical basis for future molecular targeting immunotherapy. In addition, the correlation between FDX1 and TMB and MSI proved that FDX1 closely related with the TME in human cancers. Based on the above results, it seems that FDX1 could be a viable target for anticancer therapy.

The results of our pathway analysis demonstrated that the relation between FDX1 expression level and classical tumor-associated pathways and immune-associated pathways is complex and related to tumor types. For example, our data showed that FDX1 expression level was positively associated with angiogenesis, apoptosis, citrate_cycle, DNA_repair, DNA_replication, G2M_checkpoint, P53_pathway, tumor_Inflammation and tumor_proliferation pathway in LGG, while find no relationship or even a negative association in other tumor types. The role and mechanisms of FDX1 in cell signaling needed to explored in further studies.

The challenge of drug resistance is a significant barrier to the success of preclinical and clinical cancer therapies. Our analysis of the correlation between FDX1 expression and the IC50 of over 750 anti-cancer drugs was conducted using the GSCA database. The results suggested that FDX1 expression was closely related to sensitivity of many drugs, such as PIK-93 and 17-AAG. Further validations in KIRC found that pazopanib, sorafenib, temsirolimus, axitinib and elesclomol showed a better therapeutic effect in the FDX1 high expression group. Recent studies have suggested that new treatment approaches, such as drug delivery systems in combination with elesclomol, may have improved efficacy in treating tumors^45^. Further experimental validation is needed to determine whether FDX1 can be used as a potential target and predictor for cancer immunotherapy.

Although we analyzed FDX1 through a comprehensive and systematic process, using multiple databases and R 4.1.0 to cross-verify, some limitations remain. Firstly, the sequencing and microarray data from different databases were inconsistent and lacked specificity and granularity, which could lead to systematic bias. Secondly, it necessary to conduct in vivo or in vitro experiments to determine FDX1's potential function, which would improve the credibility of our findings. Thirdly, although we concluded that FDX1 expression was associated with prognosis of human cancers and immune cell infiltration, direct evidence concerning FDX1 playing a role in immune infiltration on prognosis is still lacking. Thus, it remains unclear how FDX1 regulates immune response, and further study is needed. Fourthly, some contradictory findings regarding individual cancers were observed in our study. Therefore, it is necessary to investigate the expression and function of FDX1 further using a larger sample size. Finally, clinical trials with modulator of FDX1 have not been conducted. As a result, we do not have specific and comprehensive data that demonstrates the benefit of modulator of FDX1 in surviving cancer models or halting tumor growth. Prospective studies of FDX1 expression and its role in immune infiltration of cancer are needed in the future.

In conclusion, our results indicated that the expression of FDX1 related to patients' prognosis and immune cell infiltration in different type of cancers. Furthermore, the FDX1 gene may be a potential prognostic biomarker for tumor diagnosis and assessment. While this study is based on bioinformatics analysis, it requires further experimental validation. To confirm the prognostic value of FDX1, as well as to investigate underlying molecular mechanisms of tumor immunity, further prospective studies are required.

## Supplementary Information

Supplementary Information 1.

Supplementary Information 2.

## Data Availability

The datasets analysed during the current study are available in several online repositories (GEPIA (http://gepia.cancer-pku.cn/detail.php?gene=FDX1), TIMER2.0 (http://timer.comp-genomics.org/), BIOGPS (http://biogps.org/#goto=genereport&id=2230), KM plotter (https://kmplot.com/analysis/index.php?p=service) and UALCAN(http://ualcan.path.uab.edu/cgi-bin/TCGAExResultNew2.pl?genenam=FDX1&ctype=ACC) databases], GSCA (http://bioinfo.life.hust.edu.cn/GSCA/#/drug).
