# Supplementary figures and images for: Prognostic and immunological role of FDX1 in pan-cancer: an in-silico analysis
Source: Sci Rep. 2023 May 16;13:7926. doi: 10.1038/s41598-023-34752-1 (PMC10188527; doi:10.1038/s41598-023-34752-1)

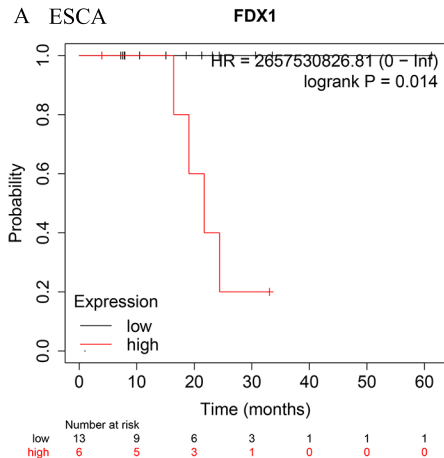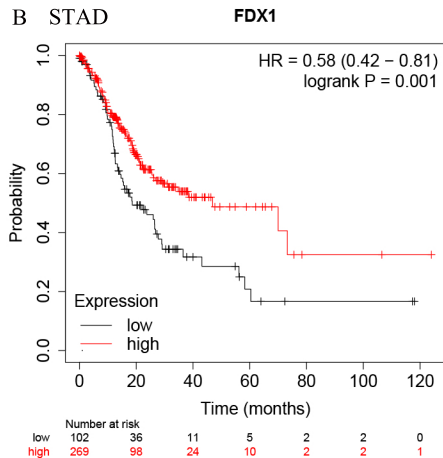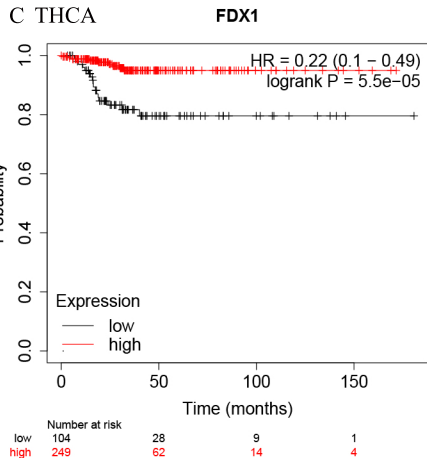

Supplement: Supplementary file 1 — Supplementary Information 1. [file 41598_2023_34752_MOESM1_ESM.pdf]
