# Supplementary material for: Prognostic and immunological role of FDX1 in pan-cancer: an in-silico analysis
Source: Sci Rep. 2023 May 16;13:7926. doi: 10.1038/s41598-023-34752-1 (PMC10188527; doi:10.1038/s41598-023-34752-1)
Supplement: Supplementary file 2 — Supplementary Information 2. [file 41598_2023_34752_MOESM2_ESM.docx]

**Supplementary figure legend**

Figure S1: Kaplan-Meier survival curve of human cancers with high and low FDX1 expression analyzed by the Kaplan-Meier plotter database.
